# Plasma-treated alginate hydrogels modulate porcine leukocyte activity *in vitro*

**DOI:** 10.3389/fimmu.2026.1750122

**Published:** 2026-08-07

**Authors:** Joanna Wessely-Szponder, Giulia Fornabaio, Dominika Nguyen Ngoc, Beata Drzewiecka, Cristina Canal

**Affiliations:** 1 Department of Pathophysiology, Faculty of Veterinary Medicine, University of Life Sciences, Lublin, Poland; 2 Biomaterials, Biomechanics and Tissue Engineering (BBT) Group, Department of Materials Science and Engineering and Institute for Research and Innovation in Health (IRIS), Universitat Politècnica de Catalunya - BarcelonaTech (UPC), Barcelona, Spain; 3 Barcelona Research Centre in Multiscale Science and Engineering (CCEM), UPC, Barcelona, Spain; 4 CIBER de Bioingeniería, Biomateriales y Nanomedicina (CIBER-BBN), Instituto de Salud Carlos III, Madrid, Spain

**Keywords:** alginate, cold atmospheric plasma (CAP), immune cell, plasma-treated hydrogel, reactive oxygen and nitrogen species (RONS)

## Abstract

**Introduction:**

This study investigates, for the first time, the effects of plasma-treated alginate hydrogels (PTH) on porcine leukocyte activity *in vitro*. Cold atmospheric plasma (CAP) generates reactive oxygen and nitrogen species (RONS), which can modulate immune cell behavior and may promote tissue repair.

**Results:**

Our findings demonstrate that PTH induce a controlled increase in nitrite and superoxide production in both neutrophil and monocyte cultures, particularly at higher treatment levels, without evidence of lytic cell death or significant loss of viability. PTH application resulted in a modest non-significant decrease in neutrophil metabolic activity without promoting degranulation, while monocyte metabolism remained unaffected. These results suggest that therapeutic doses of CAP elicit moderate oxidative stress capable of activating leukocytes while preserving their functional stability.

**Conclusions:**

Overall, PTH demonstrated low cytotoxicity toward host immune cells *in vitro*, suggesting they may be eventually employed as bioactive materials for supporting early inflammatory responses. While their potential to enhance wound healing or tissue repair remains speculative, our findings warrant further investigation for applications in both human and veterinary medicine.

## 1 Introduction

The inflammatory cascade following tissue injury is a dynamic and step-wise process, aimed at restoring the structure and function of damaged tissues (1). Neutrophils are circulating immune cells that play a crucial role in response to tissue injury and fight infections. As professional phagocytes, they employ multiple mechanisms to eliminate pathogens, including the generation of reactive oxygen and nitrogen species (RONS) during oxidative burst, as well as the release of proteolytic enzymes such as elastase and myeloperoxidase (MPO) during degranulation (1). However, excessive or dysregulated neutrophil activity can also drive chronic inflammation and contribute to non-healing wounds, conditions that often lack effective therapeutic options (1, 2). Strict regulation of the inflammatory process is therefore paramount, especially during prolonged or excessive inflammation, when neutrophils may exacerbate tissue damage by amplifying the inflammatory response and releasing toxic mediators (1, 2). A better understanding of the signals that promote neutrophil plasticity (specifically, the switch from an inflammatory to a reparative phenotype) could lead to the development of therapies that promote tissue repair while limiting excessive inflammation or fibrosis (3). RONS can be considered as omnidirectional signaling agents involved in multiple physiological processes such as cell proliferation and differentiation. Both those supplied from external sources and those generated by activated neutrophils and monocytes, act as intracellular messenger molecules that participate in the regulation of various signaling pathways. Furthermore, leukocyte-generated RONS can exert paracrine effects on other cells, such as macrophages, fibroblasts, endothelial and epithelial cells (4). While a low, controlled and sustained release of RONS is key in cell signaling and immune modulation (5–8), an excessive imbalanced RONS generation can lead to various detrimental effects, such as cell injury or neoplastic transformation (9, 10). Excessive RONS generation leads to oxidative stress and tissue damage, whereas too low levels impede the physiological cellular and molecular responses in wound healing, leading to the development of a chronic wound. Therefore, the level of RONS production must be tightly controlled for adequate tissue repair (11).

In fact, in regenerative medicine applications, a modest increase in RONS production is beneficial for the wound healing cascade, whereas a dramatic increase reduces cell adhesion and impairs platelet function (12).

Alginate hydrogels have long been used in regenerative medicine both in humans and animals for such applications as wound healing, drug delivery or tissue engineering (13–17). The encapsulation of drugs in alginate hydrogels increases their potential in various clinical applications. In recent years, hydrogels have been proposed as vehicles for RONS generated by cold atmospheric plasma (CAP) as an innovative approach that expands their medical applications (18–21).

CAPs, also known as the fourth state of matter, are partially ionized gases with promising prospects for many biomedical applications, including disinfection, wound healing, and cancer treatment. Therefore, CAPs are gaining increasing popularity in medicine, veterinary medicine, agriculture, and food science (22) and are in clinical use already for chronic wounds (23, 24), diabetic foot ulcers (25–27), or chronic venous leg ulcers (28), among others. In veterinary medicine CAP treatment was reported as an adjunct therapy for wound healing in dogs and cats (29, 30) and a pilot clinical study in cats oncology was also published (31).

Many of their therapeutical properties can be ascribed to the RONS they generate in contact with air (9, 32). CAP-treated hydrogels (PTH) are emerging as promising local delivery systems for CAP-generated reactive oxygen and nitrogen species (RONS) (33, 34). These hydrogels, composed of hydrophilic polymer networks, offer advantages such as high water content, biocompatibility, and tunable properties, mimicking natural tissue environments (35, 36). They can effectively generate, store, and release RONS, overcoming limitations of CAP-treated liquids by providing local confinement and sustained delivery to a diseased site. Current applications include their use in cancer therapies, where the RONS generated in these hydrogels have demonstrated cytotoxic potential towards cancer cells (33, 34). Furthermore, these biomaterials hold significant potential for wound healing and tissue regeneration, as hydrogels generally provide a moist environment, promote cell migration and proliferation, act as supportive scaffolds for cell growth, and can deliver bioactive agents. Their ability to regulate inflammation, prevent infection, and support tissue regeneration makes them valuable for improving outcomes in various clinical scenarios, including chronic wounds (37–39). Recent works have employed plasma-treated saline to hydrate alginate dressings which showed antibacterial effects *in vitro* and promoted wound healing *in vivo* (40).

Bekeschus et al. conducted extensive studies on the effect of CAP on different subpopulations of immune cells, indicating its role in neutrophil activation. According to other studies, CAP treatment reduced metabolic activity in peripheral blood neutrophils, whereas antimicrobial activity and viability remained intact (41). Neutrophils are not only professional phagocytes, but also crucial signaling cells that interact with other immune cells like monocytes. Consequently, modulating their functions is pivotal for regulating the early inflammatory phases of tissue healing (42).

This work aims to evaluate the response of neutrophils and monocytes to the application of CAP-treated alginate hydrogels in the context of tissue repair. Our goal was to investigate the effect of CAP on leukocytes, i.e. short- and long-term neutrophil cultures, and isolated monocytes, as well as their interactions with alginate-biomaterial for possible veterinary applications. For this purpose, we assessed leukocyte activity after stimulation with lipopolysaccharide (LPS), and we investigated the effects of PTH on the production of RONS, on the secretory response, and the survival of short-lived and long-lived neutrophil cultures, as well as blood-derived monocytes. The study was performed using porcine blood, as pigs are well-established models in regenerative and veterinary medicine, particularly for studying the treatment of infected or hard-to-heal wounds (43, 44).

## 2 Materials and methods

### 2.1 Preparation of injectable alginate hydrogels

Injectable alginate hydrogels were prepared from a 0.5% w/v sodium alginate solution (PanReac A3249, Barcelona, Spain) aqueous solution that were crosslinked under sterile conditions. Briefly, a 1 M calcium sulfate dihydrate crosslinking solution (CS) (1.021610.500, Merck, Darmstadt, Germany) and a 125 mM disodium hydrogen phosphate dihydrate retardant solution (RS) (71644, Fluka BioChemika, Buchs, Switzerland) were prepared in sterile deionized water. These two solutions were then combined at equal volumes. Immediately after mixing, the CS/RS blend was added to the alginate solution at a 1:10 volumetric ratio to initiate the ionic crosslink process. The resulting mixture was flicked to ensure homogeneity and immediately pipetted onto the dry 24-well plate.

CAP treatment of the alginate solutions before crosslinking was performed with a argon kINPen jet (NEOPLAS GmbH, Greifswald, Germany) for treatment times between 1 and 10 min, and these stored at room temperature, according to a previously described method (45). The CAP treatments were done on 1 mL of alginate solution in a 24 well plate at 1.1 standard liter per minute (slm) argon flow rate, 10 mm distance, at room temperature.

For biological essays, these CAP-treated alginate solutions were stored for seven days and then 200 μL were pipetted on the bottom of the well and crosslinked under sterile conditions. One minute later, cell suspensions of neutrophils or monocytes isolated from porcine blood were immediately poured on these hydrogels. The well plates were then incubated at 37°C with 5% CO_2_ for 1 h or 24 h.

CAP-generated reactive species were analyzed using chemical probes according to previously published methods (46, 47). Hydrogen peroxide was quantified using the Amplex Red method (48). For the preparation of 10 mL reagent solution, 50 μL of 20 mM Amplex Red stock solution in DMSO and 10 μL of 250 U mL−1 HRP stock solution in phosphate buffer (0.1 M, pH 6) was mixed with 9.940 mL of ultrapure water. Subsequently, 50 μL of Amplex Red reagent were added to 50 μL of CAP-treated solution in a black 96-well plate. After incubating the well plate at 37 °C for 30 minutes, the fluorescence was measured (λex/em = 560/590 nm) using a microplate reader (Synergy™ HTX, Biotek, Winooski, USA). Calibration lines were built using standard solutions of hydrogen peroxide prepared in the same media used during CAP treatment.

Nitrite ions were measured, using the Griess test (49). Griess reagent was prepared dissolving sulfanilamide, N-(1-naphthyl)-ethylenediamine dihydrochloride and phosphoric acid in ultrapure water to obtain 1% w/v, 0.1% w/v and 1.2% w/v concentrations, respectively. In a 96-well plate, 50 μL of Griess reagent were added to 50 μL of CAP-treated solution. After incubating the sample at room temperature for 10 minutes, the absorbance was measured at 540 nm using a microplate reader. Calibration lines were built using standard solutions of sodium nitrite diluted in the same media used during CAP treatment.

### 2.2 Neutrophil isolation and cultures

Neutrophils were isolated from porcine blood collected at slaughter, according to a previously described method (50, 51). Before the experiment, cell number and viability were assessed using the R1 Automated Cell Counter (Olympus, Warsaw, Poland). The purity of the isolated cells was confirmed by Giemsa-May-Grunewald staining (85% neutrophils). Cell viability was approximately 90%, as assessed by the Trypan blue exclusion test.

**Figure 1:**
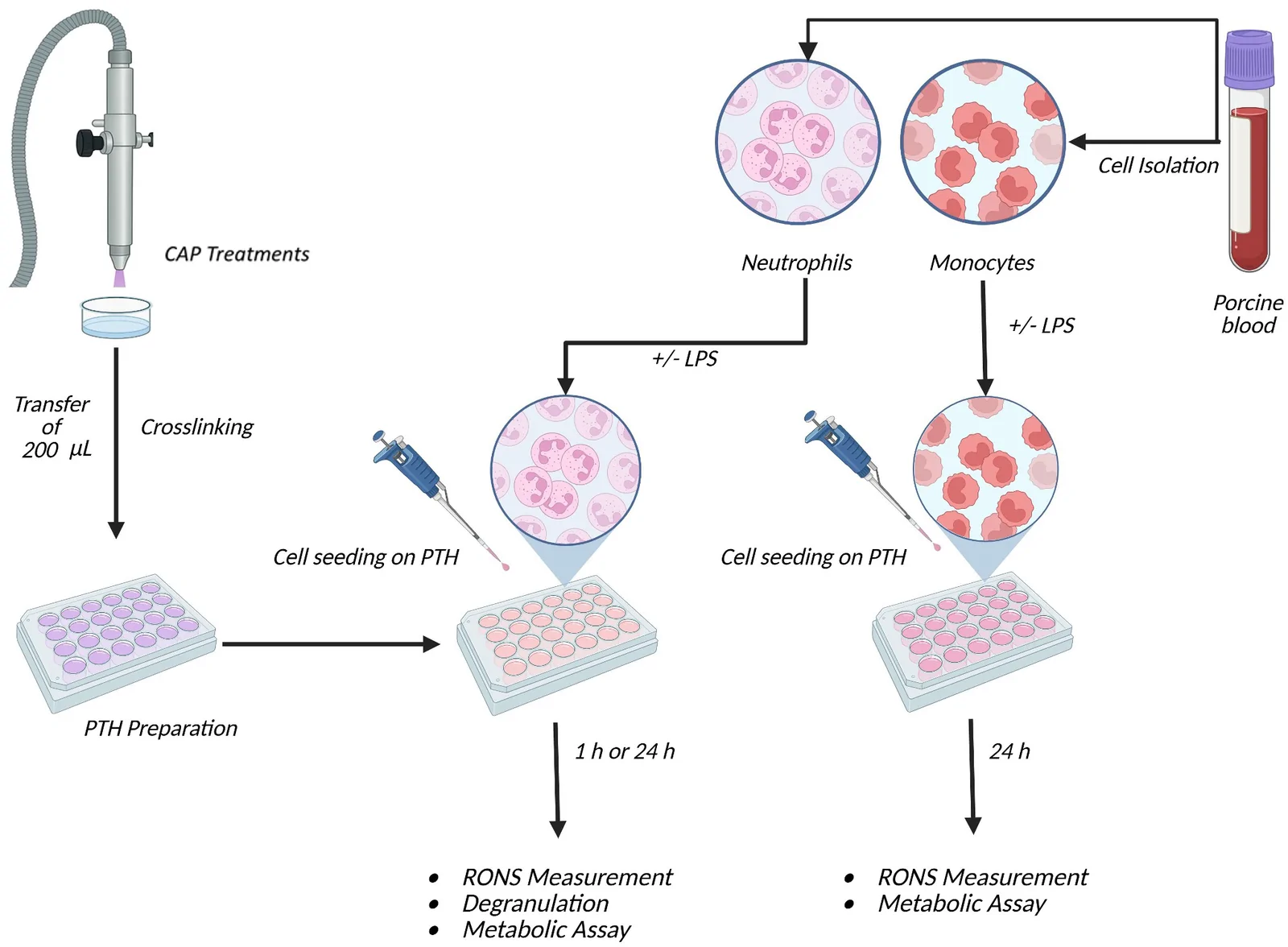
Experimental workflow: CAP treatment of alginate solutions that are transferred to 24-well plates and crosslinked to obtain PTH. Subsequent isolation of neutrophils (short-lived or long-lived) or monocytes from porcine blood, and cell seeding on the PTHs. Created with BioRender Premium.

The obtained neutrophil suspensions were used right after isolation (short-lived cultures) or after 24-h incubation at 37°C, 5% CO_2_ (long-lived cultures). The effects of PTH on these cultures were evaluated at two time-points, namely at 1 h and at 24 h. The design of each experimental setting is described below and illustrated in **Figure 1**.

#### 2.2.1 Short-lived neutrophil culture

Right after isolation from porcine blood, neutrophil suspensions were seeded at a density of 1 × 10^6^ (in 1 mL of PBS) onto 24-well culture plates containing non-treated (NTH) or CAP-treated hydrogels (PTH1-10) and incubated for 1 h or 24 h at 37 °C, 5% CO. To simulate bacterial infection, half neutrophil cultures were stimulated with LPS from E. coli (serotype 055: B5, Sigma-Aldrich, Poznan, Poland), at a final concentration of 1 μg/mL. The LPS-stimulation of neutrophil cultures occurred at the same time of PTH treatments. Neutrophil cultures without PTH or NTH treatment served as controls (marked as “w/o”).

#### 2.2.2 Long-lived neutrophil culture

Neutrophil suspensions obtained as described in section 2.2. were seeded on 24-well culture plates. To obtain long-lived cultures, neutrophil cultures were incubated for 24 h in PBS, at 37 °C, 5% CO_2_. The resulting long-lived neutrophil cultures were then transferred onto 24-well plates containing non-treated (NTH) or CAP treated hydrogels (PTH1-10) and incubated for 1 h or for 24 h at 37 °C, 5% CO_2_. As for short-lived cultures, half of these long-term cultures were stimulated with LPS during PTH treatment, to simulate a E. coli infection. Long-lived neutrophils not subjected to NTH or PTH were used as controls (marked as “w/o”).

### 2.3 Peripheral blood mononuclear cells isolation and monocyte culture

Peripheral blood mononuclear cells (PBMC) were isolated from porcine blood by Gradisol L (Aqua Medica, Lodz, Poland) density-gradient centrifugation as described previously for rabbit or human cells (50, 52). The monocyte suspension, obtained according to method described by Nadeem et al., was adjusted to 1 × 10^6^ cells/mL in DMEM without Sodium Pyruvate (Thermo Fisher Scientific, Waltham, USA) supplemented with 10% Bovine Calf Serum (BCS) and seeded onto PTH placed in 24-well culture plates. Before the experiment, cell number and viability were assessed using the R1 Automated Cell Counter (Olympus, Warsaw, Poland). The purity of the isolated cells was confirmed by Giemsa-May-Grunewald staining (around 85% mononuclear cells). Cell viability was approximately 90%, as assessed by the Trypan blue exclusion test.

As for neutrophils, half of monocyte cultures were stimulated with LPS during PTH treatment. Unstimulated or LPS-stimulated monocytes were then incubated with NTH or PTH for 24 h at 37°C, 5% CO_2_. Additional wells seeded only with cell suspensions served as controls (**Figure 1**).

### 2.4 Leukocyte activity assays

RONS generation: For this assay, the hydrogels and cells were both kept in the original wells. Two technical replicates of 50 µL of conditioned cell culture medium were collected from each well and transferred to a new 96-well plate for the colorimetric reactions. The nitrite accumulation in culture medium, as a marker of NO production by leukocytes, was measured using the Griess reaction and calculated with a standard curve of different concentration of nitrite (53, 54). Nitrite release from neutrophils or monocytes was calculated by subtracting the nitrite values obtained from NTH or PTH samples incubated without cells in culture medium from those measured in the corresponding cell cultures.

Similarly, superoxide generation of leukocytes in contact with PTH for 1 or 24 h was assessed after 15 min incubation of neutrophil cultures with 0.1% nitro blue tetrazolium solution (NBT, Sigma-Aldrich, Mannheim, Germany) at room temperature. The absorbance was then read at 545 nm, and the generation of superoxide was calculated using the extinction coefficient of NBT (21.1 nmol) (50, 51, 54). Superoxide release from neutrophils or monocytes was calculated by subtracting the values measured from NTH or PTH samples incubated without cells in culture medium from those measured in the corresponding cell-containing samples.

Neutrophil degranulation assay: The activity of porcine neutrophils was evaluated after 1- or 24-h incubation with NTH or PTH. Elastase and MPO release from azurophilic granules were assessed based on the cleavage of azocasein (Sigma-Aldrich, Poznan, Poland) or o-phenylenediamine (Sigma-Aldrich, Poznan, Poland), respectively, using a microplate reader BioTek EL800 (BioTek, Janki, Poland). Alkaline phosphatase (ALP) release from secretory granules was determined after 10 min incubation at 25 °C with substrate 4-nitrophenyl phosphate disodium salt hexahydrate (Sigma-Aldrich, Poznan, Poland) (50, 51).

### 2.5 Cytotoxicity assay

Cytotoxicity was evaluated based on the MTT metabolic activity assay, according to previously described methods (55, 56). Briefly, the neutrophil or monocyte cell suspensions from each experimental group were transferred into the 96-well microplates (CytoOne^®^, STARLAB International GmbH, Hamburg, Germany). The unstimulated cells served as control (marked as “cells”), whereas other groups marked as NTH or PTH 1–10 were test groups. 20 µL of 3-(4,5-dimethylthiazol-2-yl)-2,5-diphenyltetrazolium bromide solution (MTT, Sigma-Aldrich, St. Louis, USA, 5 mg/mL) were then added to each well and incubated 4 h, at 37°C, 5% CO_2_. After the incubation, formazan crystals were dissolved by adding 200 µL solvent (Dimethyl sulfoxide) to each well. The microplates were then shaken at room temperature for 10 minutes and the absorbance at λ_max_ =570 nm was read by a microplate reader. The percentage of metabolic activity was calculated using the formula: (Sample optical density/Control optical density) *100.

Lactate dehydrogenase (LDH) assay, based on measuring the activity of cytoplasmic enzymes released by damaged cells, was conducted using the protocol from Cold Spring Harbour Protocol (57). The assay was performed for every experimental group in each independent experiment.

### 2.6 Statistical analysis

Statistical significance was determined using one-way analysis of variance (ANOVA) followed by Tukey’s multiple comparisons test with 95% confidence. Statistical significance was reported when p < 0.05* (<0.01**, <0.001***, and <0.0001****). Figures were created using Prism GraphPad 10.5.0, BioRender Premium and Microsoft PowerPoint 2021.

### 2.7 Ethics statement

Ethical approval was not required for this study because blood samples were obtained post mortem from pigs slaughtered for food production during routine procedures at a licensed slaughterhouse. No animals were slaughtered specifically for the purposes of this study, and no experimental procedures were performed on live animals. According to Directive 2010/63/EU and Polish national legislation, including the Act of 15 January 2015 on the Protection of Animals Used for Scientific or Educational Purposes (Journal of Laws 2015, item 266), the use of post-mortem biological material obtained from animals slaughtered for food production does not require approval from an institutional ethics committee.

## 3 Results

### 3.1 Characterization of the CAP-treated alginate hydrogel

**Figure 2:**
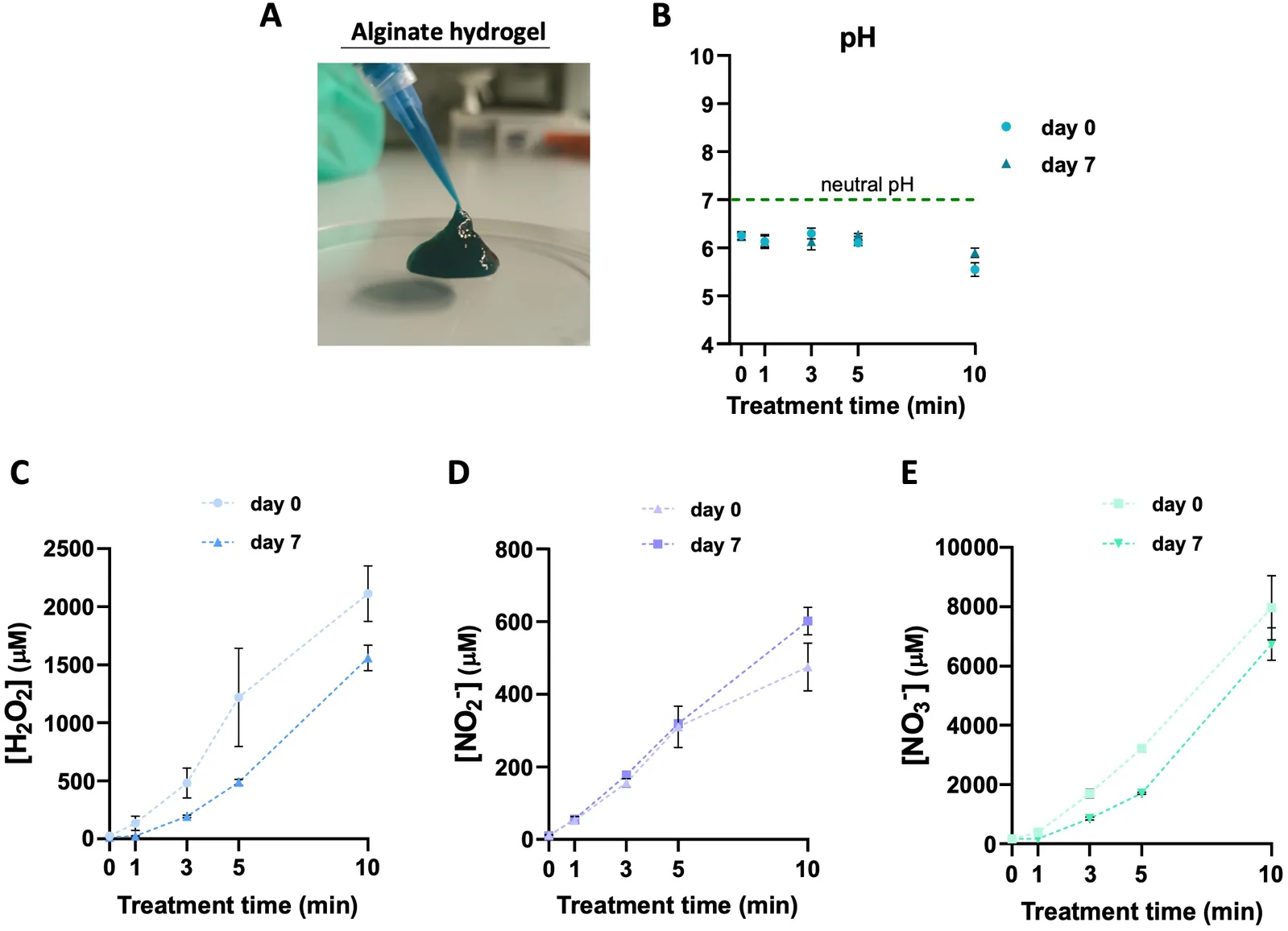
Plasma-treated hydrogel (PTH) as a vehicle for delivery of RONS. **(A)** injectability of alginate hydrogel, loaded with green food coloring for better visualization. **(B)** pH-value of plasma-treated alginate solution (PTA) for different treatment times. Concentration of H_2_O_2_ **(C)**, NO_2_^−^ **(D)**, and NO_3_^−^ **(E)** of plasma-treated alginate solution (PTA) for different treatment times. pH and RONS were measured right after the treatments and 7 days after.

The alginate hydrogels prepared are injectable, as shown by the manual extrusion tests performed using sterile syringes, and can crosslink into a stable shape afterwards. The alginate hydrogels were loaded with a green dye for better visualization (**Figure 2A**).

Before crosslinking, the alginate solution was treated with CAP for 1–10 min and three biologically relevant long-lived RONS were quantified on the same day (after 1 h) and 7 days post-treatment. The generation of hydrogen-peroxide (H_2_O_2_), nitrates (NO_3_^−^) and nitrites (NO_2_^−^) with CAP was found on the hydrogels, their concentration progressively increasing with increasing CAP treatment time, up to 10 min. (**Figures 2C–E**).

As CAP treatment is known to acidify liquids, the pH of CAP-treated alginate solutions was also measured. Alginate acted as a buffer to stabilize the pH-value around neutral even for longer CAP treatment times (**Figure 2B**), which can be beneficial for cell survival. This is relevant for the stability of RONS generated in PTA, as acidic pH favors reactions in which H_2_O_2_ is depleted.

Importantly, the levels of H_2_O_2_ and NO_3_^−^ only decay slightly one-week post-CAP treatment, while NO_2_^−^ was stable throughout the experiment (**Figures 2C–E**). Similarly, the quasi-neutral pH of the hydrogel was preserved even after one-week post-treatment.

### 3.2 Effect of PTH on short-lived unstimulated neutrophil cultures

To assess the effect of PTH on neutrophil cultures, the plasma-treated alginate was crosslinked and put in contact with neutrophils as described in **Figure 1**.

**Figure 3:**
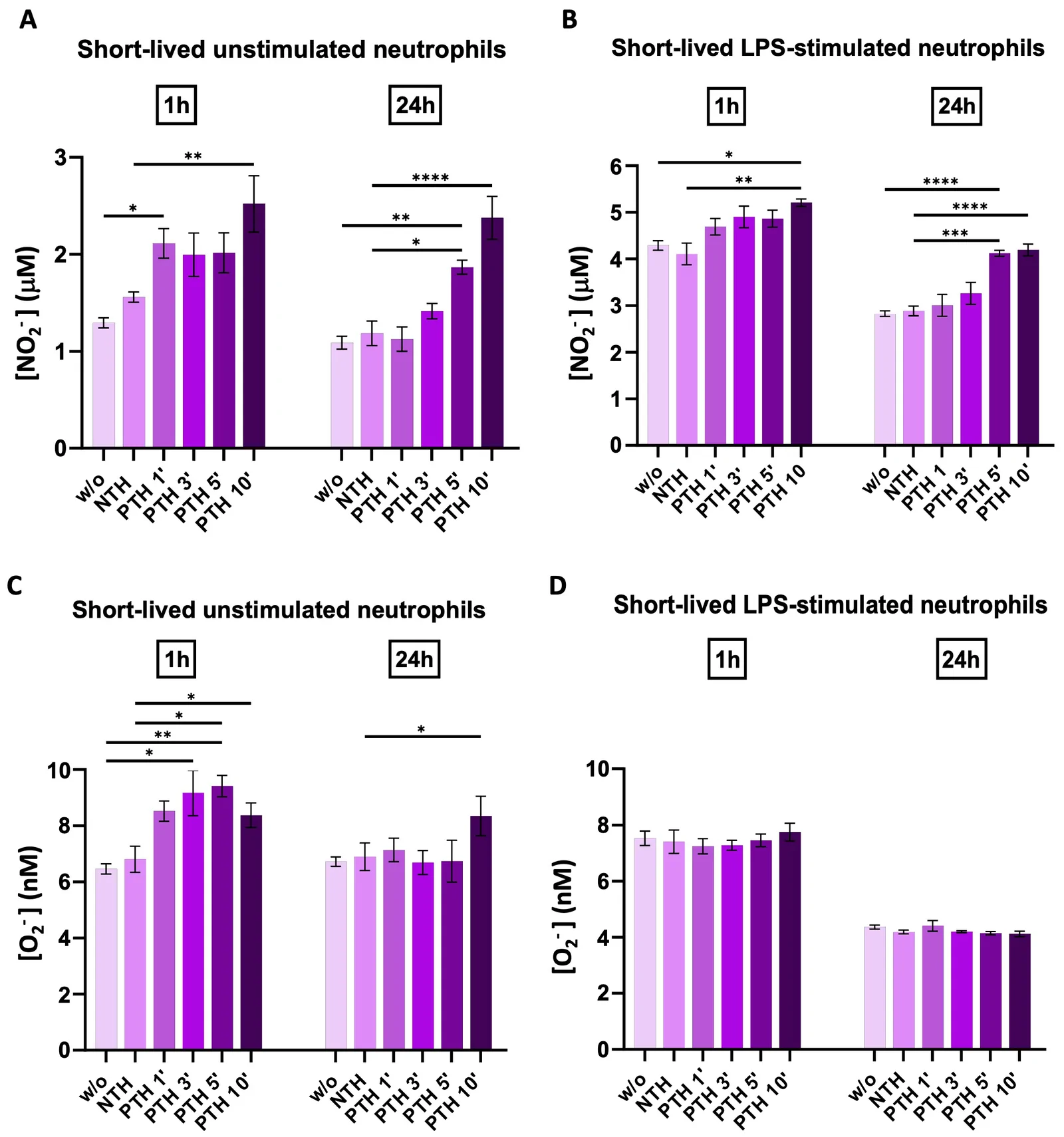
Effect of PTH on short-lived neutrophil cultures. Concentration of NO_2_^−^ released in DMEM culture medium by short-lived unstimulated **(A)** or LPS-stimulated **(B)** neutrophils, incubated with NTH or PTH 1–10 for 1 h and 24 h. NO_2_^-^ produced by cells was obtained after subtracting the concentration of NO_2_^-^in plasma-treated alginate (PTH). Concentration of O_2_^−^ released in DMEM culture medium by short-lived unstimulated **(C)** or LPS-stimulated **(D)** neutrophils, incubated with NTH or PTH 1–10 for 1 h and 24 h. w/o: cells alone, control group. (n=6, obtained with 2 independent replicates – 2 pigs and 3 technical replicates from each pig) One-way ANOVA (Tukey’s multiple comparison test) was used to determine statistical significance (p < 0.05^*^, <0.01^**^, <0.001^***^, and <0.0001^****^).

The generation of nitric oxide (NO) by the neutrophils in contact with the hydrogels was estimated based on nitrite concentrations in culture medium after treatment with NTH or PTH1-10. Nitrite concentration increased significantly (p<0.05) in short-lived unstimulated neutrophils incubated for 1 h with PTH1–10 and for 24 h with PTH3-10, if compared to control group and to NTH (**Figure 3A**).

In short-lived LPS-stimulated neutrophil cultures, the concentration of nitrite was significantly higher in comparison with unstimulated cultures (**Figure 3B**). In LPS-stimulated cultures incubated for 1 h, a significant increase in nitrite was only noted in samples treated with PTH10 (**Figure 3B**). Conversely, after 24-h incubation a significant increase in NO_2_^−^ was noted after treatment with both PTH5 and PTH 10, if compared to cells alone and treated with NTH.

Generation of superoxide increased significantly in unstimulated neutrophils treated with PTH1–10 for 1 h, while in cultures incubated for 24 h a significant increase was only registered after treatment with PTH 10 (**Figure 3C**). In short-lived LPS-stimulated neutrophils, generation of superoxide remained unchanged in response to PTH 1-10, both after 1 and 24 h incubation (**Figure 3D**).

To further demonstrate the influence of PTH on neutrophil activity, the analysis of cell degranulation was conducted. In short-lived unstimulated and LPS-stimulated cultures, no significant differences were noted in ALP release, in response to PTH1–10 after 1 and 24 h incubation (**Supplementary Figures 1A, B**). Similarly, elastase and MPO release remained unchanged in all the experimental conditions assessed (**Supplementary Figures 1C–F**).

MTT assay in unstimulated short-lived neutrophil cultures revealed unchanged metabolic activity, compared to control group (**Supplementary Figure 1G**). In line with this outcome, LDH assay did not reveal any increase of enzymatic activity, compared to cells alone (data not shown). Conversely, the metabolic activity of LPS-stimulated short-lived neutrophils showed a modest (not significant) decrease after 1 or 24 h incubation with PTH5 and PTH10 (**Supplementary Figure 1H**).

### 3.3 Effect of PTH on long-lived neutrophil cultures

**Figure 4:**
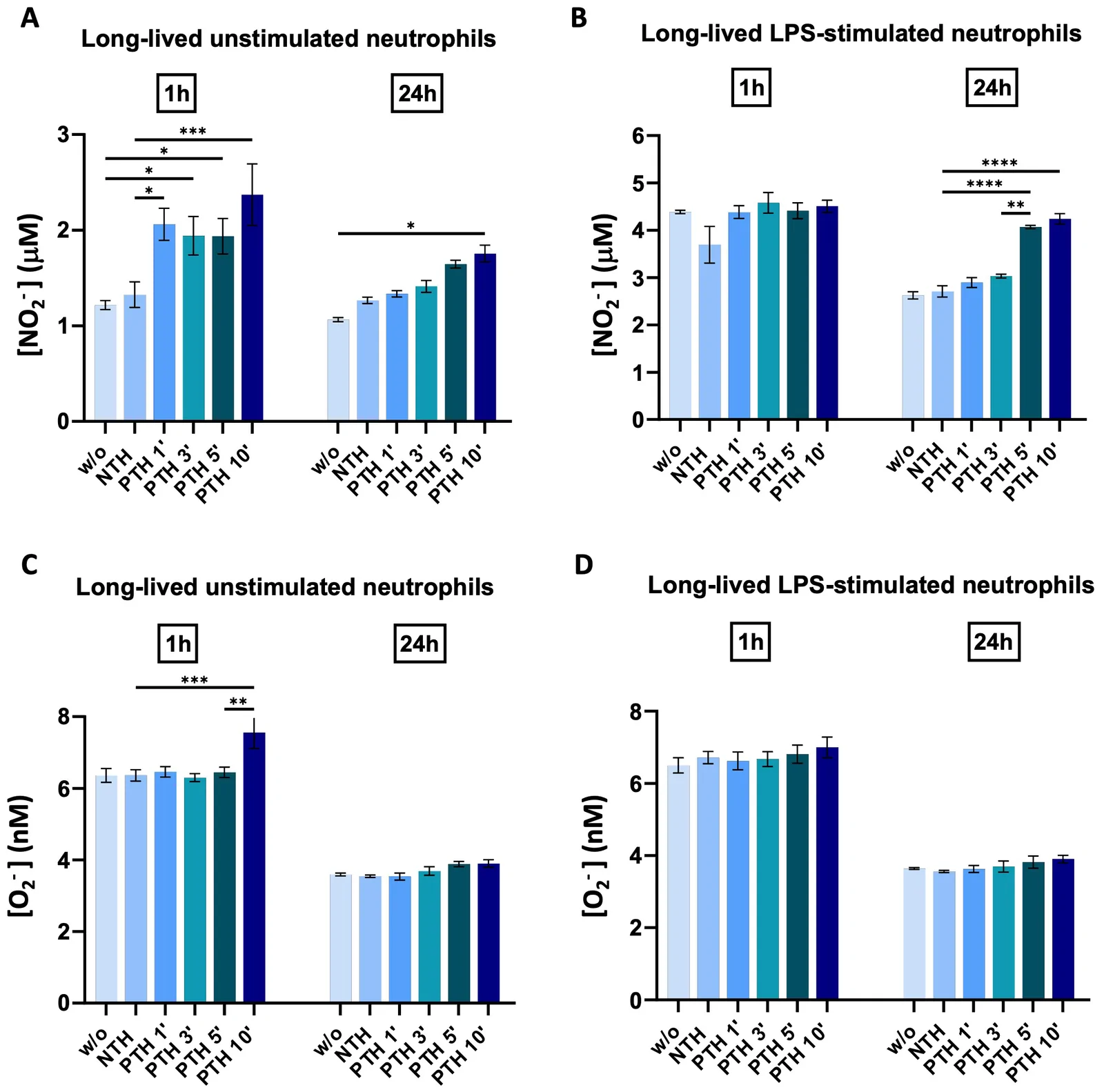
Effect of PTH on long-lived neutrophil cultures. Concentration of NO_2_^−^ released in DMEM culture medium by long-lived unstimulated **(A)** or LPS-stimulated **(B)** neutrophils, incubated with NTH or PTH 1–10 for 1 h and 24 h. Concentration of O_2_^−^ released in DMEM culture medium by long-lived unstimulated **(C)** or LPS-stimulated **(D)** neutrophils, incubated with NTH or PTH 1–10 for 1 h and 24 h. w/o: cells alone, control group. One-way ANOVA (Tukey’s multiple comparison test) was used to determine statistical significance (p < 0.05*, <0.01**, <0.001***, and <0.0001****).

The effect of RONS from PTH was also assessed in long-lived neutrophil cultures that were pre- incubated for 24 h at 37°C, 5% CO_2_, before being treated with PTH. A significant increase of nitrite concentration (p<0.05) was noted in samples treated with PTH 1–10 for 1 h, compared to cells alone and to NTH. Similar results were observed in long-lived neutrophil cultures after 24 h incubation with PTHs (**Figure 4A**). In long-lived LPS-stimulated neutrophil cultures, the concentration of nitrite was significantly higher in comparison with unstimulated cultures. However, no significant differences were observed in response to PTH treatments after 1-h incubation. Conversely, after 24-h incubation a significant increase in NO_2_^−^ was registered after treatment with both PTH5 and PTH10, if compared to cells alone or treated with NTH (**Figure 4B**).

Generation of superoxide significantly increased in unstimulated long-lived neutrophils treated with PTH10 for 1 h, while no significant increase was registered after 24- h incubation (**Figure 4C**). In long-lived LPS-stimulated cultures, generation of superoxide remained unchanged in response to PTH1-10, both after 1- and 24-h incubation (**Figure 4D**).

In both unstimulated and LPS-stimulated long-lived cultures, no significant differences were noted in ALP release, in response to PTH1–10 after 1- and 24-h incubation (**Supplementary Figures 2A, B**). Similarly, elastase and MPO release remained unchanged in all the experimental conditions assessed (**Supplementary Figures 2C–F**).

MTT assay in unstimulated long-lived neutrophil cultures revealed unchanged metabolic activity, compared to the control group (**Supplementary Figure 2G**). In line with that, LDH assay did not reveal any increase of enzymatic activity in treated samples, compared to cells alone (data not shown). Conversely, LPS-stimulated long-lived cultures showed a modest (not significant) decrease of metabolic activity in response to 1- or 24-h incubation with PTH5 and PTH10 (**Supplementary Figure 2H**).

### 3.4 Blood-derived monocytes culture

**Figure 5:**
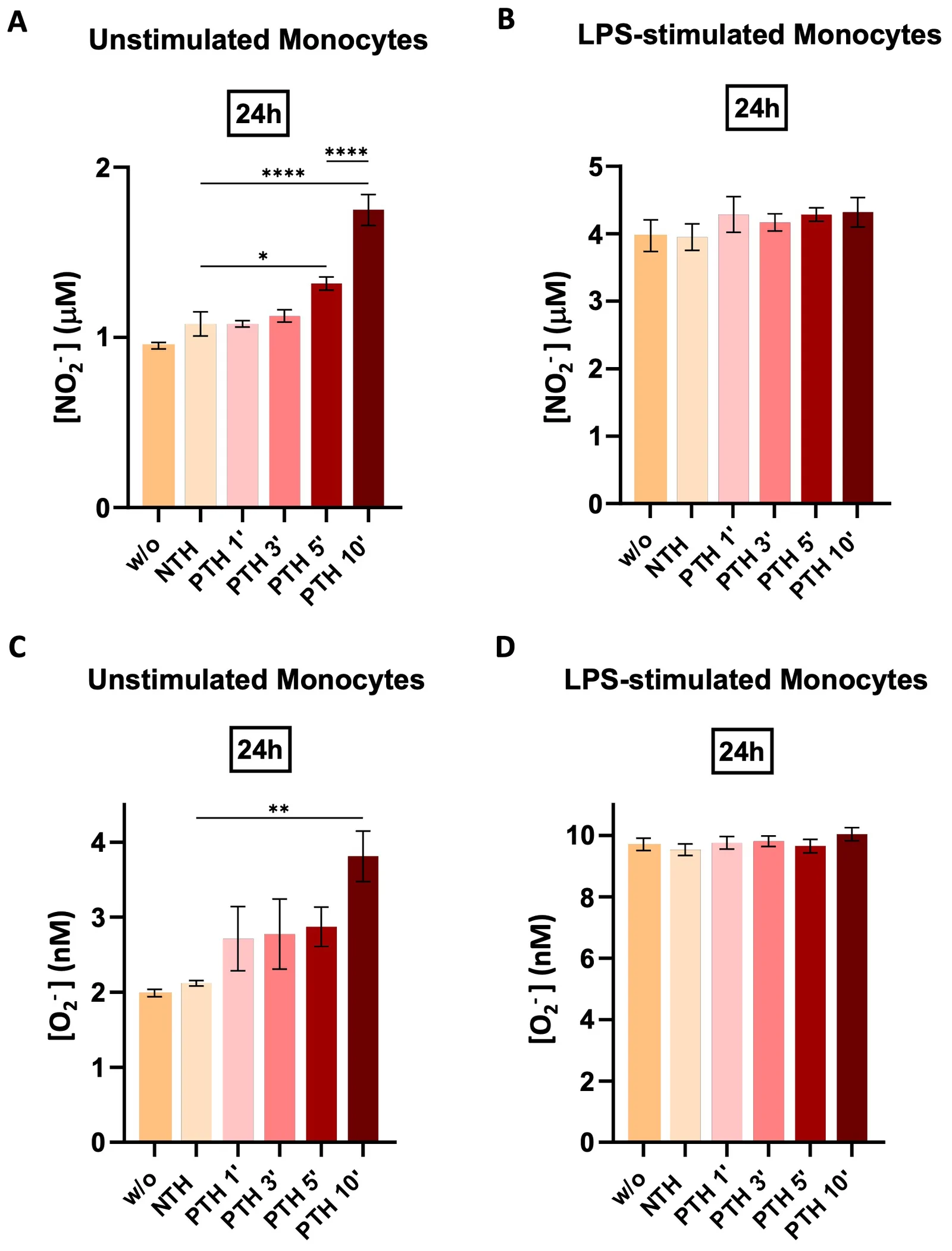
Effect of PTH on monocyte cultures. Concentration of NO_2_^−^ released in DMEM culture medium by unstimulated **(A)** or LPS-stimulated **(B)** monocytes, incubated with NTH or PTH 1-10–24 (h) Concentration of O_2_^−^ released in DMEM culture medium by unstimulated **(C)** or LPS-stimulated **(D)** monocytes, incubated with NTH or PTH 1-10–24 (h) w/o: cells alone, control group. One-way ANOVA (Tukey’s multiple comparison test) was used to determine statistical significance (p < 0.05*, <0.01**, <0.001***, and <0.0001****).

RONS generation was also assessed in blood-derived monocytes that were incubated for 24 h with PTH. Nitrite concentration was more elevated in unstimulated monocytes treated with PTH5 and PTH10, if compared with control groups (**Figure 5A**), whereas a significant increase in superoxide generation was only noted in cells treated with PTH10 (**Figure 5C**).

In LPS-stimulated monocyte cultures, RONS production was overall higher, if compared with unstimulated monocytes. Such increase in nitrite and superoxide levels masked the PTH-induced augmentation in all the LPS-stimulated samples, which showed no differences after 24-h incubation (**Figures 5B–D**).

No significant differences in metabolic activity were detected in response to PTH application in both unstimulated and LPS-stimulated monocytes (**Supplementary Figure 3**).

In line with that, LDH assay did not reveal any increase of enzymatic activity in PTH-treated monocytes, compared to cells alone (**Supplementary Table 1**).

## 4 Discussion

CAP has gained attention for its ability to generate RONS, which can modulate biological processes such as inflammation, tissue regeneration, and immune activation. When applied to biomaterials like alginate hydrogels, CAP treatment enhances their therapeutic potential by enabling the generation of RONS for local release.

PTH, particularly those based on alginate, have been primarily investigated for their antitumor and cytotoxic effects (9, 33, 45). However, their immunomodulatory properties, especially in relation to leukocyte behavior, remain underexplored and have been addressed here for the first time. The release of RONS from PTH may promote tissue repair, making them promising candidates for wound healing applications in both human and veterinary medicine (58).

The PTH employed in this work are injectable and able to accumulate RONS from CAP in a treatment-time dependent manner. Interestingly, the long-lived RONS generated therein (H_2_O_2_, NO_2_^−^ and NO_3_^−^) are stable up to one week and have shown here to modulate the activity of porcine leukocytes *in vitro*.

Neutrophils are key players in the innate immune response, capable of producing RONS and releasing proteolytic enzymes upon activation. In our study, short-lived porcine neutrophils exposed to PTH1–10 for 1 h or PTH3–10 for 24 h showed a significant increase in nitrite concentration and superoxide generation (**Figure 3**).

After activation, neutrophils can release proteolytic enzymes such as elastase to remove damaged tissues and prevent wound infection (59). However, excessive extracellular release of neutrophil-derived proteolytic enzymes after activation or lytic cell death can lead to tissue injury and may hinder wound healing (60, 61). For this reason, we assessed the response to PTH by examining the release of elastase and MPO from primary granules and ALP from secretory granules in cultured short- and long-lived neutrophils.

Despite the oxidative activity observed in neutrophils (**Figure 3**), the release of elastase, myeloperoxidase (MPO), and alkaline phosphatase (ALP) release remained unchanged (**Supplementary Figure 2**), indicating that primary and secretory granules were not disrupted. The LDH assay confirmed the absence of membrane damage (**Supplementary Table 1**), suggesting that PTH did not induce lytic cell death. These findings indicate that PTH induced changes in leukocyte oxidative metabolism without detectable degranulation or membrane disruption under the experimental conditions. However, they do not by themselves establish a beneficial or therapeutically favorable inflammatory phenotype although they might be eventually contribute to infection control and wound modulation without causing tissue injury (57, 60).

Interestingly, long-lived neutrophils, which persist under specific conditions, exhibited similar responses (**Figure 4**). No significant changes were observed in granular enzyme release or LDH levels, even when cultures were stimulated with LPS and treated with PTH5 or PTH10. A modest reduction in metabolic activity was noted, which may reflect non-lytic cell death mechanisms such as apoptosis or autophagy (**Supplementary Figure 1**). These findings align with previous reports indicating that neutrophils, as “professional phagocytes”, are highly resistant to oxidative stress and remain functionally intact under high therapeutic doses of CAP exposure (32, 62).

Monocytes treated with PTH also showed increased nitrite and superoxide production, with LPS stimulation further amplifying this response (**Figure 5**). However, MTT and LDH assays revealed no significant changes in metabolic activity or viability, confirming the absence of cytotoxic effects.

Monocytes, known for their role in inflammation and tissue repair, appear highly tolerant to CAP-induced oxidative stress. Their intrinsic antioxidant capacity likely protects them during RONS-mediated activation, allowing them to contribute to immune modulation without undergoing damage (32, 62).

Various forms of ROS, including superoxide, play a crucial role in the wound healing process. In addition to their antimicrobial activity, they function as secondary messengers in signaling cascades, participating in the regulation of chemotaxis, angiogenesis, and such processes as cell growth and migration or the deposition of extracellular matrix (ECM) components. Strict regulation of the ROS levels and the timing of their production is essential to ensure their effective action. Excessive concentrations of the superoxide or impaired detoxification mechanisms lead to oxidative stress, whereas insufficient ROS levels inhibit a range of cellular and molecular processes (dependent on ROS-mediated signaling) necessary for wound healing, ultimately resulting in the formation of chronic wounds. Consequently, interest in the role of ROS in wound healing and their potential therapeutic application has grown significantly in recent years. However, further elucidation of the precise signaling pathways and mechanisms through which ROS participate in the wound healing process is required (11).

Previous studies demonstrated that Nitric oxide (NO) not only controls essential neutrophil functions such as chemotaxis, adhesion, ROS production, pathogen killing, and apoptosis, but also induces neutrophil differentiation and granulopoiesis. Human neutrophils produce NO at a rate comparable to the one of endothelial cells (63, 64). In a study on patients with chronic granulomatous disease, a significant increase in NO levels was found in cultured human neutrophils after the addition of IFN-γ *in vitro* (65). Nitric oxide generation by peripheral blood neutrophils almost doubled in patients with allergic asthma, in response to allergens and a-IgE antibodies (66). Moreover, previous research of our group indicated that NO generated by leukocytes isolated after an injury in rabbit (50) or ovine (67) models was higher compared to control animals.

Given the importance of the porcine model in research on various pathological conditions, immune system responses have previously been analyzed on porcine leukocytes in contexts such as oxidative stress (68) and interactions of the host cells with biomaterials (51). Furthermore, Singleton et al. evaluated porcine monocyte-derived macrophages and dendritic cells using *in vitro* systems; these authors conducted phenotypic and functional analyses of both sub-populations and subsequently used them to assess immunological potential (69). Recently, the antibacterial and antifungal immune responses of pigs have also been investigated in the context of wound healing, utilizing burn and excision wound porcine models treated with CAP (70). Interestingly, even relatively small changes in the production of RONS as reported here may reflect altered leukocyte activation, as these mediators typically act within low micro- or nanomolar concentration ranges.

Previous research has shown that, in response to external stimulation caused by CAP-induced RONS, activated phagocytes generate oxidative stress with the release of endogenous RONS (71, 72). Additionally, CAP-induced activation of endothelial NO synthase contributes to acute wound repair by regulating cell migration, proliferation, and differentiation, as demonstrated in a mouse burn model. Treatment of muscle tissue with CAP-generated RONS has been shown to stimulate a transient yet robust inflammatory response that enhances angiogenesis and immune activation, promoting more effective tissue repair (73). Thus, CAP has shown promise in treating chronic wounds, infected lesions, and diabetic ulcers (74, 75). These works employed direct CAP treatment on the cells or wounds (and thus the effects could be ascribed to any of the multiple components of CAP – electromagnetic fields, ions, electrons, UV-VIS radiation and RONS). However, no prior works have assessed the effects of delivering the RONS generated by CAP through hydrogels, which open a number of possibilities for application in wound healing, and the high stability shown in this work opens many options for future clinical application.

Following PTH application, we observed increased superoxide and nitrite generation, without evidence of lytic damage. Controlled endogenous RONS generation by host immune cells has potential therapeutic relevance, particularly when it does not cause harmful oxidative stress on surrounding tissues. Such RONS generation may contribute to the regulation of inflammatory responses through auto- and paracrine signalling (4, 11, 76). To our knowledge, this is the first study to evaluate the effects of CAP-treated alginate hydrogels on porcine leukocytes, demonstrating that higher therapeutic doses of PTH increase RONS generation without inducing cell lysis.

While our *in vitro* model does not allow the direct assessment of intracellular RONS, the extracellular measurements and functional assays demonstrate that PTH modulates leukocyte oxidative activity without detectable cytotoxicity. Although these findings support the biological activity of PTH and its apparent safety under the experimental conditions, additional functional studies are required to determine whether these changes translate into beneficial immunomodulatory effects.

A primary limitation of this study is the exclusive reliance on an *in vitro* model, which prevents the evaluation of complex, *in vivo* intercellular interactions. Additional mechanistic analyses could shed light on why we observed a modest decrease in MTT reduction, without a corresponding increase in LDH leakage. Some of these could include the measurement of intracellular ROS generation, the assessment of cytokine production, and the phenotypic characterization of leukocyte activation states. Also, assessing non-lytic cell death mechanisms, such as apoptosis or autophagy could also be of interest in future works.

In all, plasma-treated alginate hydrogels represent a promising platform for immune modulation and tissue repair. By modulating leukocyte oxidative activity without inducing detectable lytic cell damage *in vitro*, PTH may represent a promising platform for tissue repair and immune modulation. Further *in vivo* studies are needed to validate these findings and explore their clinical applicability.

## 5 Conclusions

The results of this study illustrate, for the first time, that CAP-treated alginate hydrogels can modulate porcine leukocyte activity *in vitro*. We demonstrated that PTH application generates mild oxidative stress without increased degranulation in porcine neutrophil cultures. No lytic damage and only a moderate decrease in metabolic activity were also reported in both short-lived and long-lived neutrophil sub-populations. We also demonstrated that longer CAP-treatment times cause increased RONS generation in monocyte cultures, without affecting their viability. The effect on intercellular signalling remains to be investigated, as the low endogenous release of RONS may influence various paracrine pathways. Overall, our findings demonstrated that therapeutic doses of CAP show low cytotoxicity for the host white blood cells *in vitro*, suggesting that PTH may eventually be employed as bioactive materials for supporting the first phases of the inflammatory response. PTH represent a promising experimental platform for further investigation in immune modulation and regenerative medicine.

Our study could therefore pave the way for a more in-depth characterization of the effects of CAP and PTH in modulating immune responses.

## Data Availability

The raw data supporting the conclusions of this article will be made available by the authors, without undue reservation.
