# Supplementary material for: Plasma-treated alginate hydrogels modulate porcine leukocyte activity in vitro
Source: Front Immunol. 2026 Aug 7;17:1750122. doi: 10.3389/fimmu.2026.1750122 (PMC13493507; doi:10.3389/fimmu.2026.1750122)
Supplement: Supplementary file 1 [file Supplementaryfile1.docx]

**Supplementary material**

**Plasma-treated alginate hydrogels modulate porcine leukocyte activity *in vitro*.**

*Joanna Wessely-Szponder^a†*^, Giulia Fornabaio^b,c†^, Dominika Nguyen Ngoc^a^, Beata Drzewiecka^a^, Cristina Canal^b,c,d*^*

***Supplementary Figure 1****. ALP released by short-lived unstimulated A) or LPS-stimulated B) neutrophils, incubated with NTH or PTH 1-10 for 1 h and 24 h. Elastase released by short-lived unstimulated C) or LPS-stimulated D) neutrophils, incubated with NTH or PTH 1-10 for 1 h and 24 h. MPO released by short-lived unstimulated E) or LPS-stimulated F) neutrophils, incubated with NTH or PTH 1-10 for 1 h and 24 h. Metabolic activity of short-lived unstimulated G) or LPS-stimulated H) neutrophils, incubated with NTH or PTH 1-10 for 1 h and 24 h. w/o: cells alone, control group.*

***Supplementary Figure 2****. ALP released by long-lived unstimulated A) or LPS-stimulated B) neutrophils, incubated with NTH or PTH 1-10 for 1 h and 24 h. Elastase released by long-lived unstimulated C) or LPS-stimulated D) neutrophils, incubated with NTH or PTH 1-10 for 1 h and 24 h. MPO released by long-lived unstimulated E) or LPS-stimulated F) neutrophils, incubated with NTH or PTH 1-10 for 1 h and 24 h. Metabolic activity of long-lived unstimulated G) or LPS-stimulated H) neutrophils, incubated with NTH or PTH 1-10 for 1 h and 24 h. w/o: cells alone, control group.*

***Supplementary Figure 3****. Metabolic activity of unstimulated or LPS-stimulated monocytes, incubated with NTH or PTH 1-10 for 24 h. w/o: cells alone, control group.*

***Supplementary Table 1****. LDH release from neutrophils stimulated with PTH (% of control) after 24 h incubation*

| Cell culture | w/o | NTH | PTH1’ | PTH3’ | PTH5’ | PTH10’ |
| --- | --- | --- | --- | --- | --- | --- |
| Short-lived unstimulated neutrophils | 100 | 98.5±0.7 | 99.00±1.4 | 98.5±0.7 | 99.5±0.7 | 99.5±0.7 |
| Short-lived LPS-stimulated neutrophils | 100 | 99.5±0.7 | 99.5±0.7 | 99.0±0.5 | 99.5±0.5 | 99.5±0.3 |
| Long-lived unstimulated neutrophils | 100 | 98.5±0.7 | 99.5±1.4 | 99±1.4 | 100±0.4 | 99.0±0.6 |
| Long-lived LPS-stimulated neutrophils | 100 | 98.5±0.7 | 99±1.4 | 99.0 | 98.5±0.7 | 99.5±0.7 |

***Supplementary Table 2.*** *LDH release from monocytes stimulated with PTH (% of control) after 24 h incubation*

| Cell culture | w/o | NTH | PTH1’ | PTH3’ | PTH5’ | PTH10’ |
| --- | --- | --- | --- | --- | --- | --- |
| Unstimulated monocytes | 100 | 98.5±0.7 | 99.0±1.4 | 99.5±0.7 | 98.5±0.7 | 103.5±2.1 |
| LPS-stimulated monocytes | 100 | 99.0±1.4 | 99.0±0.3 | 100±0.5 | 99.0±1.4 | 106.5±0.7 |
